# Comparison of nutrition care services for adult obesity at primary care in two different periods in Saudi Arabia

**DOI:** 10.1186/s12875-023-02094-6

**Published:** 2023-07-21

**Authors:** Elham A. Aljaaly, Nahlaa A. Khalifa

**Affiliations:** 1 Clinical Nutrition Department, Faculty of Applied Medical Sciences, King Abdulaziz University, Jeddah, Saudi Arabia; 2 Respiratory Therapy Department, Faculty of Medical Rehabilitation Sciences, King Abdulaziz University, Jeddah, Saudi Arabia

**Keywords:** Adults, Dietitian, Nutrition care, Obesity, Primary care, Service evaluation, Survey

## Abstract

**Background:**

Dietitians are healthcare professionals with potential roles and impacts in primary care (PC) settings when applying knowledge and expertise to educate healthcare clients at all levels and treat chronic diseases. This study seeks to compare and evaluate the nutrition care services and practices in obesity management in primary care centres for two periods (2016 and 2019) in Jeddah city, Saudi Arabia.

**Methods:**

Surveys with service self-reporting evaluation used responses from 18 and 27 centres in 2016 and 2019. Services evaluation used no reference to a standard, but 18 of the surveyed PC centres in 2019 were previously visited in 2016 to assess the provided nutrition care services for adult obesity in PC centres. The re-visit survey investigated improvements in services achieved in 2019 concerning services for adults with obesity. A chi-square test was used to compare the surveys' results in the two periods, which resulted in a significant difference in the provided nutrition care services for adult obesity between 2016 and 2019.

**Results:**

Dietitians' employment has significantly changed (*P* < .0001) in 2019 compared to 2016. Dietitians were significantly noticed as the primary source of nutrition information after their integration into the services (*P* < .0001) in 2019. Services provided for adult obesity increased significantly (*P* value < .0001) in the second phase. However, there was no significant difference in serving adult groups between the two periods (*P* = .056).

**Conclusions:**

Integrating dietitians into the PC services significantly enhanced their role in supporting PC services for conditions relating to adult obesity, which allowed them to be the most important source of the delivered nutrition information to patients. The employment rate of PC dietitians accredited by the governing body is significantly increasing; the Saudi Commission for Health Specialties will need to monitor it to ensure that dietitians have the qualifications and skills to provide professional medical nutrition therapy to patients. Further research to evaluate the quality of PC dietetic practice and improvements in patient outcomes is required to strengthen the importance of integrating registered dietitians into the services.

**Supplementary Information:**

The online version contains supplementary material available at 10.1186/s12875-023-02094-6.

## Introduction

In Saudi Arabia, obesity has been demonstrated as a public health problem affecting both adult genders, with a total prevalence of 35% (*n* = 1419) [1]. However, soon after, another published data showed less prevalence of obesity among adult males and females, which was 21.7% (*n* = 4709) among adults ≥ 18 years old [2]. Nutrition care practices performed by health professionals to support patients in improving their dietary behaviours are used to evaluate primary care (PC) providers' nutrition competency [3]. Therefore, primary healthcare centres as settings for obesity control were addressed as one of the key attributes to tackle the problem in the Arab gulf states, with documented efforts on nutrition and physical activity [4]. The same study evaluated the available Saudi strategies, policies, and challenging programs for tackling obesity compared to the available programs in the other Arab gulf states and reported fewer efforts done by Saudi Arabia. In contrast, Saudi national, institutional and individual programs were conducted through the primary healthcare centres to tackle obesity. For example, two collaborated programs (the Diet and Physical Activity Program and Obesity Control Program) were conducted in collaboration between the Ministry of Health (MoH) and the Ministry of Education. Both programs aimed to control obesity early and to increase the recognition of obesity risk factors [5]. Other examples included randomized clinical trials conducted in primary health care settings in different areas of Saudi Arabia, including Jeddah city, which showed encouraging outcomes in achieving clinically significant weight loss of 1 to 2 pounds per week [6, 7].

In order to find tools to help the practising health professionals to manage and prevent obesity among the Saudi population, evidenced-based Saudi guidelines for obesity that were adapted from other international guidelines were pressed to healthcare clinicians in the Kingdom of Saudi Arabia in 2016 to effectively prevent and manage overweight and obesity among all age groups [8]. The Saudi guidelines have called for healthcare teams to include specialists from several disciplines, counting registered dietitians (RDs). The guidelines have also identified RDs' particular responsibilities in helping patients to change their behaviours, modify their lifestyles and manage their weight while implementing the guidelines. Although the guidelines were published in 2016 and were recommended to be used by clinicians from all professions and at all levels of health care, there needs to be more information about applying these guidelines. In 2021, a single study was conducted to evaluate the primary care physicians’ use of the Saudi obesity guidelines. Reports showed that only 9% of 234 participating physicians believed in the guidelines' role in reducing the risk of obesity [9]. Additionally, Saudi dietitians need to improve their expertise and competencies in understanding nutrition-related guidelines and policies to enhance their interaction with the multidisciplinary patient-centred team, as reports from bachelor graduates dietitians showed deficiency in skills related to understanding and writing the national and international regulations for food and nutrition [10].

Conducting descriptive service-evaluation studies to evaluate providing nutrition care services and nutrition therapy in the obesity scope is significant [11].

The quality assessment of the provided nutrition care as part of the health services and outcomes of these services on adult obesity might be complex because other factors such as patients’ readiness to change, level of compliance to educational sessions and treatment may influence the provided services. Moreover, if health professionals are particular about the need and importance of these services, their confidence in the RD's role in managing their patients could affect the outcomes of these services. However, thus far, assessing and reassessing the nutrition care services for adults with obesity in PC is vital in determining whether the services have improved, and the role of an RD as one of the PC providers is well-defined in managing the problem, particularly after the publication and the call for clinicians to follow a defined guideline. However, to ‘’the best of the authors’ knowledge,’’ no previous research was conducted in Saudi Arabia to evaluate the nutrition care services and the primary care dietetic practice concerning adult obesity. Through this project, the authors aimed to conduct a comparative study of the nutrition care services for adult obesity at primary care centres (PCCs) in Jeddah city, Saudi Arabia, in two periods. First, the study compared abstracted responses from a previously conducted survey that evaluated general provided services, including nutrition services for the adult group and those with obesity in 2016 on 18 ministries of health PCCs 'An additional file shows the unpublished data in details [see Additional file 1]', with the data of a revisit survey to 27 centres in 2019. The study hypothesized that the nutrition care services for adult obesity in the second period of 2019 have improved compared to those provided in the first period in 2016, particularly after integrating dietitians into the services and after the publication of the Saudi guidelines in preventing and managing obesity in 2016 and the recommendation to be used by clinicians using a multidisciplinary approach.

## Materials and methods

**Fig. 1:**
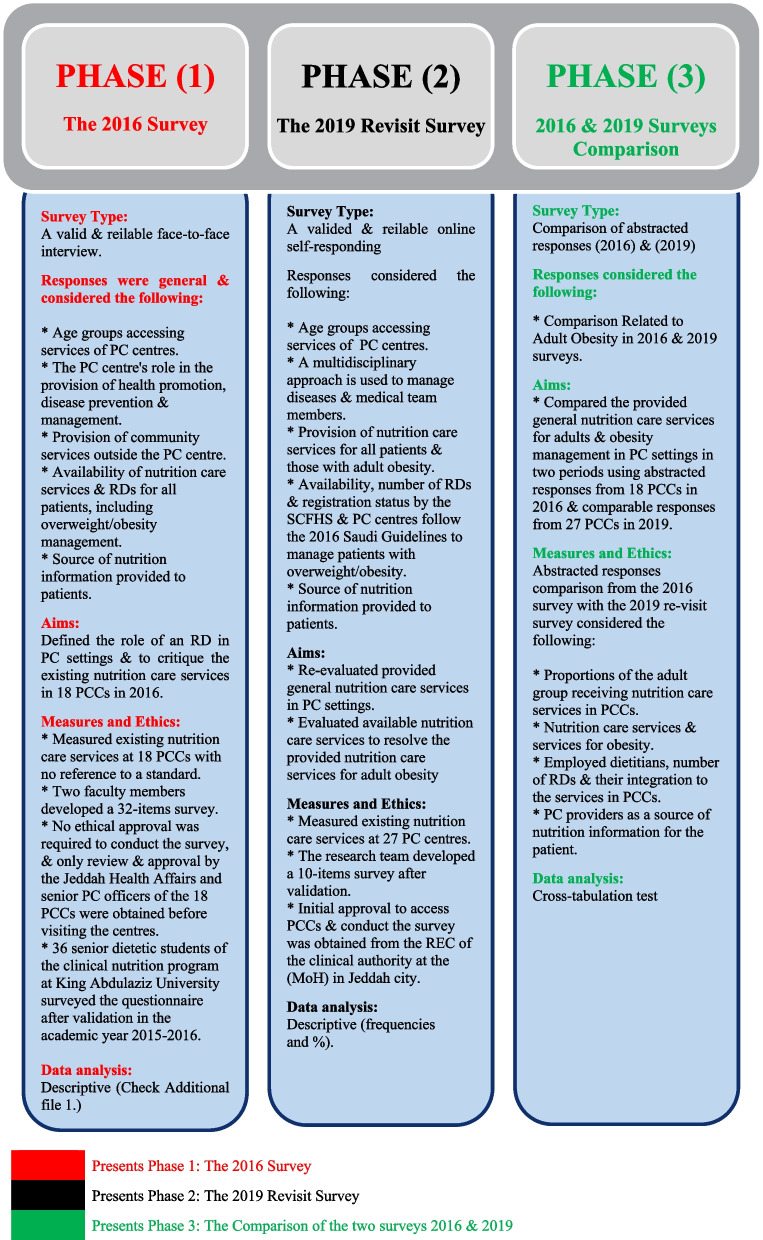
Phases of service-evaluation for nutrition care at PCCs. Figure presents phases of service evaluation for nutrition care at PCCs

This conducted study includes three phases **(**Fig. 1).

## Phase 1: The service evaluation survey in 2016

The study is a face-to-face interview survey to evaluate the provided general services and practices of disease nutrition management and the dietitian’s role in the PC setting. Senior dietetic students at King Abdulaziz University conducted the survey as part of the course related to their professional skills. The project aimed to train students in the PC setting and to enhance their competencies in measuring the provided nutrition care services for all patients with all age groups.

Please "see Additional file 1" for details of survey development, validation, reliability, and data collection and results.

## Phase 2: The re-visit services evaluation study in 2019

The research team conducted a re-visit service evaluation study in 2019 to re-evaluate the services of the PCCs concerning obesity management. A structured questionnaire was used.

## Study design, development and validation of the survey and data collection

Study type: A cross-sectional service evaluation study used an electronic self-reporting survey.

## Development and validation of the implemented survey

The authors of this study formed a 10-item questionnaire survey to evaluate the nutrition care services provided to patients regarding the involvement of RDs and services provided for adult obesity. The existing literature guided the development of survey questions [11, 12].

Responses to the survey considered the integration of RDs into the general practice team and services for obesity management, unified practices and the use of the evidenced-based Saudi guidelines for preventing and managing adult obesity. Moreover, the responses considered whether the management of patients’ weight status is conducted using a multidisciplinary approach (patient-centred), employed dietitians, how many dietitians are in the PCC and the number of accredited and registered by the Saudi Commission for Health Specialties (SCFHS) in case RDs are to be considered within the PC providers, the use of the published and recommended for use by clinicians 2016 Saudi guidelines by the PCC to manage obesity, the existence of a separate Nutrition Care Services/Department. Response structure included close-ended and categorical data (nominal and ordinal) with two or more categories.

For survey validation and testing, the authors selected seven members of an expert panel who were nutrition care providers in three general hospitals to review, edit, and approve the survey questions. Additionally, to increase the precision of the developed survey, the questionnaire testing was taken by three RDs from the same hospitals before implementation in PCCs.

The validation and testing process included the following:

### The Pre_testing phase

Before testing the survey, the expert panel members reviewed the survey items in its English version. Instruction for each question was given a score on a scale from 1 to 5 to evaluate each questionnaire item’s relevance, precision, well-pronunciation and understandability. An explanation for each criterion was provided to members of the expert panel.

### The validity conduct phase

The panel reviewed the 10-items of the survey in terms of relevance, precise, well-pronounced, and understanding for each question out of 5 scores (1- not at all relevant, 2- not relevant, 3- neutral, 4- relevant, 5- very relevant). If the score of any of the items is less than or equal to 3, then the reviewer will add a comment to the authors in that item to revise and modify accordingly. Additionally, three additional face validity questions were added to the end of the survey to test items further if they measured what it is intended to measure. Questions are testing the:1. completeness of the content, “Do you agree that the survey items encompass the most important aspects to evaluate nutrition care services for adult obesity at PC level? [Yes/No]”; “If “NO, which aspects would you incorporate?”.

2. Comprehensibility, “Do you agree that this survey items are sufficiently and coherently worded? [Yes/No]”; “If “No, which items you do not agree with their comprehensibility?” 3. Time to complete “Is the time needed for filling in the questionnaire appropriate? Use a scoring on a 0–10 scale to rate it, with 0 representing “unacceptably long” and 10 “completely acceptable”.

### The results of the conducted validity

As determined by the validity scores, all items were relevant, precise, well-pronounced, and understandable. Further evaluation showed that none of the item's content needed to be completed was not comprehensive or took a long time to be completed. Therefore, the panel approved the questionnaire, and it was ready for testing.

### Testing of the survey tool

The tool was tested face-to-face on 5 participants in their workplace: three primary care administrators and two RDs, to ensure its reliability before applying it in the survey. The research team interviewed participants on the same survey tool two weeks later. Based on this testing, all ten questions were answered similarly by participants.

## Study site, sample selection and data collection

In 2016, the first phase of this service evaluation study was conducted by senior dietetic students as part of practice training in PC settings. A face-to-face interview survey was used to collect data from senior primary care officers of 18 ministries of health PCCs covering all geographical areas of Jeddah city with permission from Jeddah Health Affairs, who selected the centres based on their participation agreement. The Health Affairs permission also included an onsite visit to the selected PCCs. In 2019, the research team revisited the same 18 centres in 2016. As a result, nine additional centres were included in the second phase. They were selected from all geographical areas of the city based on recommendations of the Health Affairs for their provision of obesity services and willing to cooperate with the research team in replying to the survey. Therefore, a total of 27 governmental-operated PCCs in Jeddah city were cross-sectionally surveyed for nutrition care services evaluation and dietetic practice for obesity. Based on most of the respondents' preferences and busy schedules, the authors obtained responses from primary care provider heads, nutrition services heads and RDs at the 27 selected PCCs using an electronic self-report survey.

## Phase 3: Data for responses’ comparison between the first and second visit (2016 & 2019)

In order to compare data for the 2019 re-visit with data from 2016 to evaluate the nutrition care services for adults with obesity, the authors conducted data analysis for the 2016 survey using descriptive analysis for all responses. Then, they abstracted the responses, which demanded to be compared with data from the second visit in 2019. The comparison aimed to evaluate services after integrating RDs into the PC services. The preliminary results of this comparison study were presented at the American Society of Nutrition “Nutrition 2020” conference, and the abstract was published [13].

The measurement of the achieved services included comparing previously assessed services in 2016 for adults in the scope of obesity management with those provided in 2019. The compared provided services data in 2016 and 2019 considered services for adults, the presence of dietitians, source of nutrition information among the medical team and the proportions of each age group receiving nutrition care services in each centre. Other comparisons included the general provided nutrition services and services for obesity.

## Statistical analysis

### The 2019 re-visit survey data analysis used descriptive analysis

After data collection, capture and cleaning, SPSS for windows (version 22) was used for analysis. The analysis described the existing nutrition care services in 2019 at 27 PCCs in Jeddah city. Variables were expressed as frequencies and percentages and summarized in tables.

### Responses’ comparison between the 2016 and 2019 surveys

Data analysis for comparisons included selected responses from 2016 and 2019 surveys conducted using a chi-square test. In addition, regrouping of responses for the two questions about the served age groups by the PC centre and the nutrition care providers was made in both surveys before conducting the test. This was made to better achieve the study outcomes in measuring the differences in the PC providers' performance for the obesity nutrition services before and after integrating the RDs into PC services and following the publication of the 2016 Saudi guidelines for clinicians. Therefore, abstracted items from the 2016 results concerning the presence of dietitians, source of nutrition information among the PC providers and the proportions of each age group receiving nutrition care services in the centre were compared with the 2019 related data.

The authors considered the STROBE checklist as guidance to confirm a clear and thorough reporting of the conducted work strategy and findings [14].

## Results

### The 2016 survey’s abstracted responses

Table 1 shows the abstracted responses from the 2016 survey on 18 PCCs in Jeddah city to be compared with related responses to the 2019 re-visit survey. The results showed that PCCs provided health care services for adults; 11.1% (*n* = 2) were grouped with the elderly group, and 61.1% (*n* = 11) were included with all other groups. In addition, only 38.9% (*n* = 7) provided nutrition care for weight problems (obesity/overweight). None responded to the question concerning employment 94.4% (*n* = 17) PCCs had employed clinical dietitians (RDs). Most centres provided nutrition care services by 94.4% (*n* = 17). The dietitian was not employed. Therefore, she/he was not the source of nutrition information, and nutrition counselling in PCCs is carried out by other PC providers such as nurses, physicians, social workers and health educators. "See Additional file 1 for all 2016 survey results".

**Table 1:** 2016 Survey’s Abstracted Responses (*n* = 18 PCCs)

Variables name	FRQ	%
**Age groups accessing the PCC’s services**		
** > *age groups are regrouped (Response* = *18)***		
Paediatrics (Infants, Toddlers, Children & Adolescents)	5	27.8
Adults (adults & elderlies)	2	11.1
All groups (all age groups served)	11	61.1
**Do you provide nutrition care services for patients?** ***Response (18 centers)***		
Yes	17	94.4
No	1	5.6
**Do the provided nutrition & dietetics services and counselling include weight management (obesity/overweight)?** ***Response (15 centers)***		
Yes	7	38.9
No	11	61.1
**Within the organizational structure of your PCC, is the dietitian or nutrition/dietetics services part of it?** ***Response (18 centers)***		
Yes	13	72.2
No	5	27.8
**Are there any nutritionists /dietitians working in your PCC?** ***Response (17 centers)***		
Yes	0	0.0
No	17	94.4
**If yes, who is the source of nutrition information?**		
Dietitians	0	0.0
Physicians	5	27.8
Nurses	5	27.8
Physicians & Nurses	3	16.7
Physicians, Nurses & Health educator	4	22.2
Social workers	1	5.6

^*^Abstracted responses from PCCs (*n* = 18) in 2016

^*^n (%) shows data presented as numbers and percentages

### The 2019 survey results

(Table 2**)** The re-visit to 27 PCCs in 2019 showed that nutrition care services at 24 PCCs are provided for the adult group when grouped with the elderly group (11.1%) and (77.8%) when counted with all other age groups. About 74% (*n* = 20) of the centres use a multidisciplinary approach to manage patients’ health status. Around 85% (*n* = 23) of the centres provide nutrition services for patients with obesity, and 44.4% (*n* = 12) of these centres follow the Saudi guidelines when managing obesity. Dietitians are employed in 85.2% of the PCCs (*n* = 23), and the SCFHS accredited all the employed ones. In about 84% of these centres, dietitians are the primary sources of nutrition information provided to patients.

**Table 2:** 2019 Survey’s Descriptive Results (*n* = 27 PCCs)

Variables name	FRQ	%
**Age groups for patients following up in the PC centres**		
** > *one esponse to the question(age groups are regrouped)***		
Paediatrics (Infants, Toddlers, Children & Adolescents)	3	11.1
Adults (adults & elderly)	3	11.1
All groups (all age groups served)	21	77.8
**Medical Team Members (Professionals) at PC centres** ***(*> *one response to the question)***		
Physicians	17	29.8
Health Educators	11	19.3
Dietitians	15	26.3
Nurses	8	14.0
Social workers	6	10.5
**Does the centre provide nutrition services?** ***(Response* = *27)***		
YES	25	92.6
NO	2	7.4
**Does your centre use a multidisciplinary approach in managing patients' diseases?** ***(Response* = *27)***		
YES	20	74.1
NO	7	25.9
**Does the centre provide nutrition services for Obesity?** ***(Response* = *26)*** 23 (88.5%)		
YES	23	85.5
NO	3	11.2
**Does your centre follow the 2016 Saudi guidelines to manage obesity?** ***(Response* = *27)***		
YES	12	44.4
NO	15	55.6
**Are there dietitians in the centre?** ***(Response* = *27)***		
YES	23	85.2
NO	6	22.2
**Are all employed dietitians registered with SCFHS?** ***(Response* = *27)***		
YES	22	81.5
NO	5	18.5
**How many dietitians are in the centre?** ***(Response* = *27)***		
Three or more RDs	5	18.5
< three RDs	22	81.5
**What are sources of nutrition services?** ***(Response* = *27)***		
Dietitians	22	81.5
Physicians	4	15.4
Nurses	1	1.0

^*^Responses from PCCs (*n* = 27) in 2019

^*^n (%) shows data presented as numbers and percentages

### The comparison data for the 2016 and 2019 surveys

**Fig. 2:**
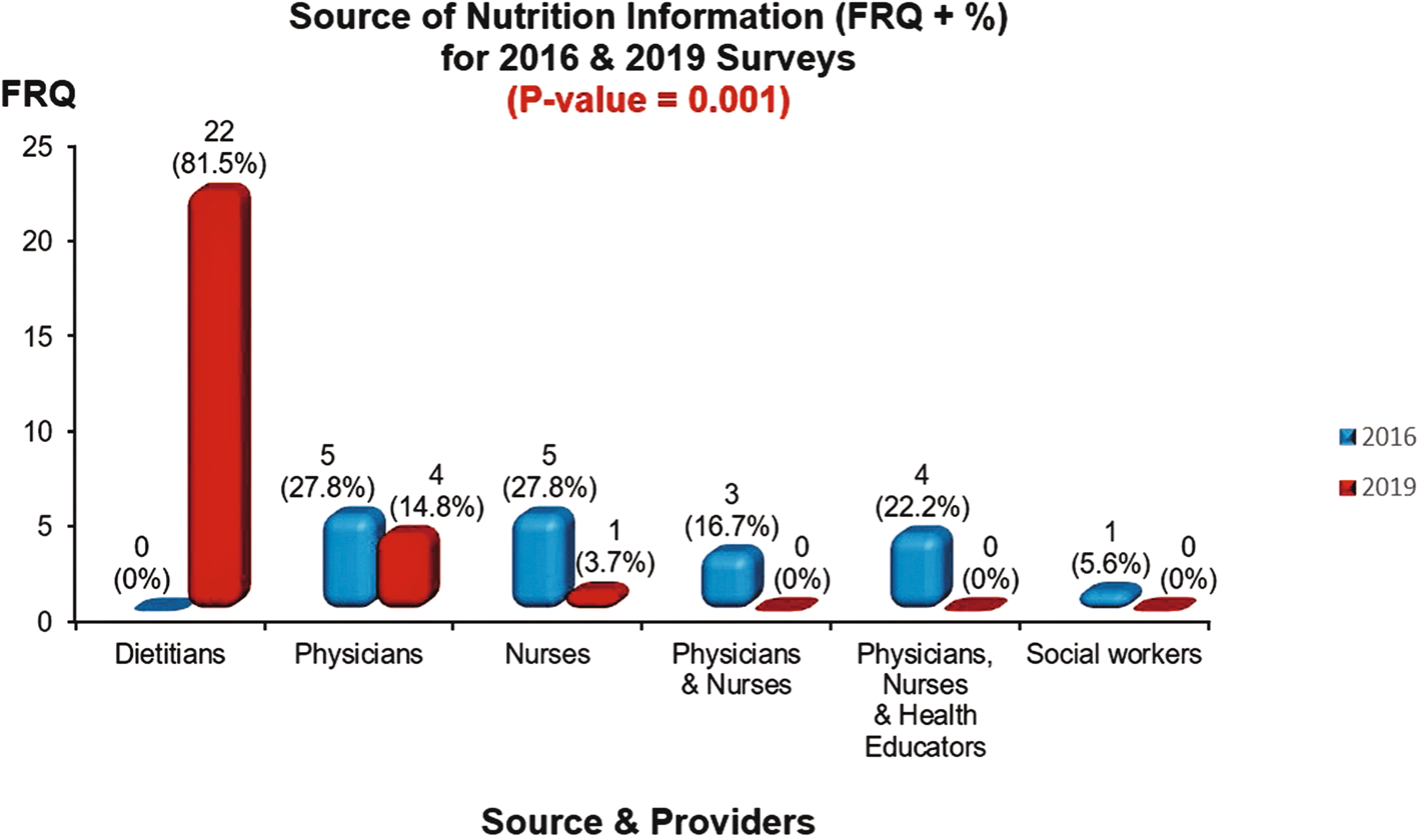
Source of Nutrition Information for 2016 & 2019 Surveys. *Bar graph showing the main source of nutrition information for patiens among primary care providers in two periods: 2016 & 2019. *Significant difference in provision of nutrion information, as the RD was mainly the nutrition information provider in 2019, compared to 2016 (*p* < 0.05)

**Fig. 3:**
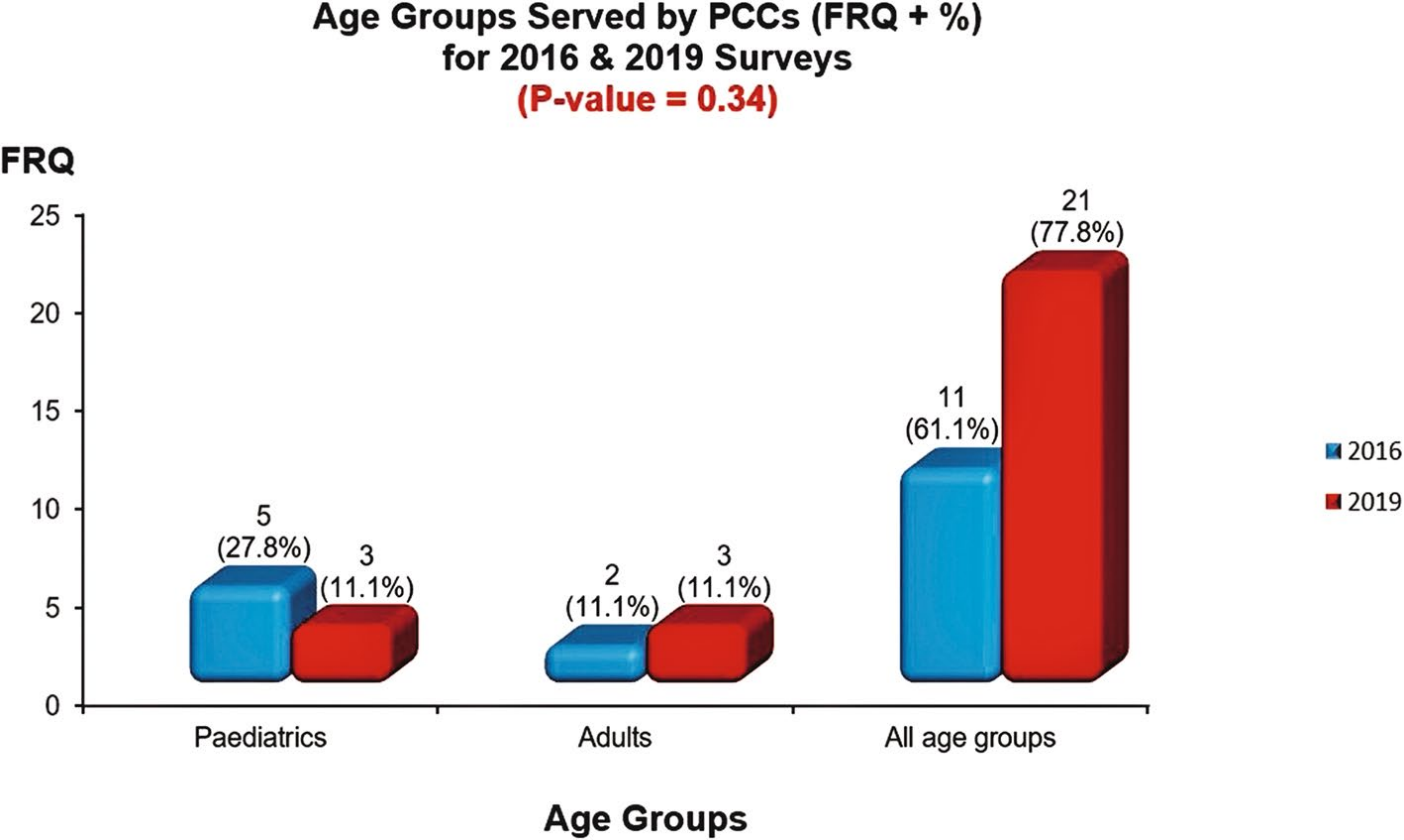
Age Groups Served by PCCs for 2016 & 2019 Survey. Bar graph showing the served age groups by PCCs in the two periods. *No significant difference in the provided services in 2019, compared to 2016 (*p* > 0.05)

Based on the conducted survey in 2016 and the re-visit survey for nutrition care services evaluation in 2019 in Jeddah city for PCCs concerning obesity management and after the integration of the RD into the services in 2019, using a cross-tabulation test for comparison **(**Table 3), results showed some significant differences in the provided nutrition care services in 2019, when compared to 2016. The employment of dietitians in PCCs has significantly increased compared to the situation in 2016 (*P* < 0.0001), and all working dietitians were registered with the SCFHS. The 2019 re-visit survey showed a highly significant change in considering health professionals as the primary source of nutrition information in the centres as dietitians were the primary source of nutrition information (*P* value < 0.0001) compared to the situation in 2016 **(**Fig. 2). Services provided for patients with obesity significantly (*P* value < 0.0001) increased in 2019 compared to 2016. More provision (92.6%, *n* = 25) for general nutrition care services was provided in PCCs in 2019. However, it did not reach a significant level compared to the report in 2016 (94.4%, *n* = 17). No significant change in the provided services for each age groups (Fig. 3).

**Table 3:** Responses comparison from the 2016 & 2019 surveys

	**Year of survey**				
**Variable**	**2016** ***N* = 18**		**2019** ***N* = 27**		***P* value**
	**FRQ**	**%**	**FRQ**	**%**	
**Age groups served by the center**					0.34
Paediatrics	5	27.8	3	11.1	
Adults	2	11.1	3	11.1	
All age groups	11	61.1	21	77.8	
**Does the center provide nutrition care services?**					0.807
Yes	17	94.4	25	92.6	
No	1	5.6	2	7.4	
**Source and provider of nutrition information**					0.001
Dietitian	0	0	22	81.5	
Physicians	5	27.8	4	14.8	
Nurses	5	27.8	1	3.7	
Physicians & Nurses	3	16.7	0	0	
Physicians, Nurses & Health educator	4	22.2	0	0	
Social worker	1	5.6	0	0	
**Does the center employ clinical dietitians?**					0.001
Yes	0	0	23	85.2	
No	17	100	4	14.8	
**Are the employed dietitians registered with the SCFHS**					
Yes	NA		23	100	
**Dose the center provide nutrition care for patients with obesity?**					0.001
Yes	7	38.9	23	88.5	
No	11	61.1	3	11.5	

^*^P-Value is significant at < 0.05

## Discussion

This cross-sectional service evaluation survey mainly aimed to compare the nutrition care services for adult obesity in PCCs located in Jeddah city within the time frame from 2016 to 2019. The study’s findings are discussed in the context of the availability of nutrition care services and dietetic practices concerning managing adult obesity using PCCs’ responses to surveys in the two periods. The detailed analysis for comparison is abstracted adult obesity-related responses to a survey conducted in 2016 and the administration of an online short 10-item questionnaire in 2019.

To facilitate dietetic students’ knowledge and competence development, take on responsibility for practice and improve nutrition services in a setting, they should be supervised, and their training should involve a series of activities [15, 16]. In this study, students’ practice supervision was integral in evaluating the delivery of nutrition services in the PC setting and reflection on the services provided by the senior dietetic students in the academic year 2016–2017 (see additional file for details). Furthermore, the obtained knowledge from the supervised project was analyzed and used as part of a broader project by comparing its data concerning adult obesity with the latest data in 2019, which identified the provided nutrition services and dietetic practice for obesity management in primary care settings in Jeddah city in the two periods.

Dealings with the national health care system require individuals, their families and the community to start at the primary level of care. PC creates the first element of a continuing healthcare process since it brings healthcare as close as possible to where people live and work, strengthening the whole health system [17, 18]. The study results showed that primary care providers delivered services to all age groups, including adults, and no significant changes were noticed in these services between the study periods concerning the adult group.

In 2016, the Saudi Arabian government, based on the proposed 2030 Saudi Vision and in collaboration with the WHO, reformed its primary care system to meet international standards in controlling noncommunicable diseases and patients with chronic diseases, which made it more recognized and accessible by 70% of the Saudi population [19].

Saudi guidelines for obesity management advice, including RDs and nutrition care at the primary care level, were also reported [8, 20]. The responses to the 2019 re-visit survey showed that about 93% of PCCs were providing general nutrition care services, and 89% of these services targeted adult obesity.

Although 2016 published comprehensive guidelines for obesity prevention and management, which were recommended to be used by a multidisciplinary team of clinicians, including dietitians for all age groups, less than 50% of the surveyed centres in 2019 reported applying and following the guidelines when managing obesity. The 2016 survey data did not include the response to this question, as the 2016 guidelines were not published then.

Saudi physicians' understanding of clinical nutrition discipline and nutrition education is limited, and the recommendation to hire dietitians in health care settings as an integral part of a multidisciplinary team to deliver therapeutic care services was noted [21, 22]. PC providers perceive that they are more trained than other health professionals in other health settings, such as nurses, to discourse weight management in PC settings [23]. However, PC providers are advised to use an integrated multidisciplinary approach to tackle the problem of obesity by managing it earlier in primary care settings [24]. The approach could support patients socially and improve interprofessional work between medical and administrative teams [25]. Dietitians' success in preventing and treating obesity was reported and confirmed by recently published reviews [26]. In addition, adult obesity management registered dietitians were reported to be responsible for enabling care to individuals looking for nutrition care services [27].

The 2016 Saudi guidelines for clinicians to prevent and manage obesity also emphasized integrating primary care providers, including the RD, in caring for adults with obesity [8].

The study confirmed that 74% of Jeddah's visited PCCs in 2019 were using a multidisciplinary team approach to managing obesity, and integrating dietitians into the primary care services was significantly improved in 2019 compared to 2016. The study also reported that dietitian is the primary source of nutrition information provided to patients in most (82%) visited centres in 2019, compared to the 2016 responses as physicians and nurses were the primary sources of nutrition information.

The employment rate for dietitians in PPCs was about 85% in 2019, compared to zero employment in 2016, who were all accredited by the governing body, the SCFHS compared to surveyed registration status for dietitians in Jeddah private and governmental-sponsored general hospitals was only (80.0%, n = 8 out 10) [12], and in other scopes of practice such as bariatric surgery (93.8%, n = 30 out of 32) [28]. The high registration rate of PC working dietitians could be explained by the current well-regulated registration by the SCFHS, as only registered clinical dietitians are allowed to manage patients nutritionally. On the other hand, the employment of non-clinical dietitians in Saudi hospitals has been the situation for three decades, where registered clinical dietitians were mainly employed from other countries, and Saudi general dietitians who are non-clinical based and graduated from the college of home economics were allowed to be employed in hospitals to provide nutrition care for patients [29]. However, the registration status has improved since the establishment of dietetic education in Saudi Arabia. Based on requirements for the Ministry of Education and SCFHS, the dietitian should hold a health program certificate, complete an internship and be registered by the governing body [30]. Therefore, it is important to note that the governing body should monitor the education and registration status of employed dietitians as current reports showed that dietitians who were educated in Saudi Arabia and practising the application of international guidelines "the Nutrition Care Process" in managing nutrition-related diseases were only reported by 79% of dietitians who were holding a Bachelor's degree in nutrition and dietetics [31].

- The potential focus of healthcare in Saudi Arabia is to implement strategic management for professional practices, which requires continuous training and education programs that target principals and practitioners [32]. Therefore, the study endorses the conduct of onsite or online workshops for health professionals concerning the need to implement evidence-based guidelines for the prevention and treatment of obesity among all age groups and adults in specific to tackle the problem and emphasize the importance of tackling the problem based on the Saudi 2030 vision and evidence-based guidelines.

## Study limitations

The following are the study limitations:

- Compared to research, the conduct of service evaluation was reported not to generate evidence as it is not compared to a standard, and they might not need ethical approval as for research studies [11]. However, in the present study, students learned and enhanced their competencies in conducting research as the same needed skills for research were applied to evaluate care in a PC setting. Although service evaluation could not generate new evidence since it was not compared with a standard, the re-visit survey in the present study generated the first visit to PCCs in 2016 as the standard for the 2019s visit. Furthermore, the MoH’s requirement to obtain ethical approval from the research ethics committee made no difference between the conduct of the service evaluation study and other research.
- No sample size calculation was performed as the researchers had to only consider the primarily selected by the Health Affairs PCCs, who agreed to allow researchers to conduct the first phase of this service evaluation survey. In 2019, the research team had to revisit the same PCCs to re-evaluate their previously evaluated services concerning adult obesity, which was the outcome measure of the study in the second phase. Therefore, when conducting further service evaluation surveys in the PC setting, it is recommended to calculate a sample size based on the total number of available centres and consider identifying a sample selection criterion.
- The authors consider that the conducted data in phase two in 2019 to evaluate PC’s moderately old nutrition services and possible change and improvement in the PC dietitians’ employment and practice of obesity management are highly expected, particularly when integrating RDs into PC providers. This boosts the authors to call for the importance of conducting a third visit to PCCs, randomly selecting PCCs to assess the provided nutrition services for obesity, particularly during the COVID-19 pandemic, to evaluate its impact on the provided nutrition services for obesity in this setting.

## Conclusions

Conducting service evaluation surveys to evaluate the nutrition care services and the impact of integrated dietitians into the primary care services was achievable and informative to evaluate services provided in the scope of adult obesity.

The study highlighted that the provision of nutrition care services concerning adult obesity has much improved in the ministry of health primary healthcare centres in 2019, compared to services in 2016. When part of the multidisciplinary primary care team, dietitians became the primary source of nutrition information delivered to patients.

The study also emphasises and brings the attention of the policy/decision-makers to the importance of integrating RDs into the multidisciplinary PC team to tackle the problem of obesity and make prevention possible in the PC setting. Moreover, for the best quality of the provided nutrition care and similar to other international policies and local settings of practice in health settings, strict rules for only employing registered dietitians to provide clinical-based services to patients and ongoing assessment of the employed dietitians’ licences are mandatory in the provision of safe, operative, unbiassed, and quality practice in adult obesity management in the setting. Furthermore, employers should conduct this since clinical dietitians are demanded to be accredited by SCFHS.

Since the study confirmed that incorporating nutrition care services into primary care could enhance a collaborative patient-centred approach to primary care management of obesity and power all involved health practitioners, including RDs, to tackle the problem in Saudi Arabia. Therefore, the research team has conducted workshops for primary care providers and planned to test the implementation of an intervention program to tackle adults' obesity problem who receive PC services and use a multidisciplinary approach based on the 2016 Saudi guidelines.

Future service evaluation studies are necessary to evaluate the quality of nutrition care services, the employment rate for RDs in primary care, and its impact on the provided nutrition care services for adult obesity.

## Supplementary Information

**Additional file 1.** 

## Data Availability

Data and details related to the 2016 survey are included as an additional file "see Additional file 1". Any other datasets related to this study or its analysis are kept with the corresponding article's author and will be provided upon request.

## Abbreviations

KAU: King Abdulaziz University
NTP: The Government National Transformational Plan
PC: Primary Care
PCC: Primary Care Centers
RD: Rigstered Dietitians
SCFHS: Saudi Commission for Health Specialties
