# Supplementary material for: Comparison of nutrition care services for adult obesity at primary care in two different periods in Saudi Arabia
Source: BMC Prim Care. 2023 Jul 21;24:153. doi: 10.1186/s12875-023-02094-6 (PMC10362681; doi:10.1186/s12875-023-02094-6)
Supplement: Supplementary file 1 — Additional file 1. [file 12875_2023_2094_MOESM1_ESM.docx]

**Additional file 1**

File name: **Additional file 1**

Title of data: The data for the 2016 Survey

Description of data: methods and results of the conducted Survey in 2016.

**The data for the 2016 Survey**

The following sections of this unpublished work contain details of the conducted survey in 2016 on 18 Primary Care Centers (PCCs) in Jeddah city and the data's descriptive results.

**Section A: Study Design, Development and Validation of the Survey and Data Collection and Analysis (Table A)**

The study is a cross-sectional service evaluation study, which used a face-to-face interview survey that KAU senior dietetic students conducted in the academic year 2015-2016 under course instructors (the authors of the present work) supervision with permission from Jeddah Health Affairs to access 18 PCCs in Jeddah city to conduct interviews and onsite visits to the selected PCCs.

This student’s project is part of the practical work for a course titled professional skills in dietetics. First, students as groups (two students for each centre) visited the 18 PCCs and used the same survey interview questionnaire. Next, each group reported the answers to their conducted interview questionnaire and observations about the visited PCC by the end of the visit. Finally, each group of students presented their work in class and provided recommendations to improve the nutrition services for the visited centre. The course instructors then provided a concluding report to the Jeddah Health Affairs with a general recommendation concerning their feedback about the existing services and recommendations to improve them.

Based on the literature, two-course instructors developed a 32-Item questionnaire survey to train senior dietetic students (n=36) on practising the dietitian role in primary care settings through evaluation of services and practices of disease nutrition management. Hence, to enhance their competencies in measuring the provided nutrition care services for patients.

An expert panel of eight faculty members from the clinical nutrition department assessed the survey items in terms of precision and value for validation, proving all items' validity. Two RDs from the KAU Hospital answered all questions to test the questionnaire and confirmed its clarity and appropriateness.

The survey was conducted using a face-to-face interview with senior primary care officers of 18 PCCs covering all geographical areas of Jeddah city. **The survey questionnaire addressed primary care resources and services in the centres, availability of RDs and their integration within the medical team when managing diseases.**

The visit to PCCs also included an onsite rotation in each PCC, evaluation of services and looking at available records and used forms for nutrition care and management such as assessments and documentation notes related to the nutrition services.

The data analysis for the survey used descriptive analysis, and variables were expressed as frequencies and percentages, and SPSS for windows (version-22) was used for analysis.

**Section B: The Results of the 2016 Conducted Survey**

The study's results (**Table B)** showed a comprehensive evaluation of the provided nutrition care services at 18 PCCs in 2016. This study used interviews and onsite visits for data collection from the selected PC settings.

Findings showed that while PCCs provide nutrition management for a few nutrition-related diseases, no formal nutrition care services are assigned for all PCCs. In all centres, there are no RDs involved in nutrition care services. Health care providers in PC centres incoherently use nutrition care services. Nurses and physicians (27.8%) are equally the primary source of nutrition information. In the absence of dietitians, other PC providers assess patients’ nutritional status, measure anthropometry, and calculate body mass index.

**Table A The Survey Questionnaire: Services provided by PCCs in Jeddah City**

| **Intrview Questionnair** | | | |
| --- | --- | --- | --- |
| **Please answer each of the questions below;** | | | |
| **Date of interview: ___/___/______** | | | **Name of interviewers: ………………..** |
| **A. CENTRE INFORMATIONS** | | | |
| 1. **Name of the primary care centre (PCC)** | | | |
| …………………………………………………………… | | | |
| 1. **Areas where the PCC is located** | | | |
| North | | | |
| South East | | | |
| South West | | | |
| Centre | | | |
| Other areas | | | |
| 1. **What is the role of your (PCC)?** | | | |
| Health promotion and disease prevention | | | |
| Intervention and treatment | | | |
| Both | | | |
| I do not know | | | |
| 1. **Do you provide community-based services outside your PCC?** | | | |
| Yes | No | I do not know | |
| 1. **Is your PCC accessed by the majority of:** | | | |
| Saudi | Non- Saudi | Both | |
| 1. **Which age group are the most accessed by your PCC?** | | | |
| Infants | | | |
| Toddlers | | | |
| Children | | | |
| Adolescents | | | |
| Adults | | | |
| Elderly | | | |
| 1. **Is each medical team, including dietitians in the centre, required to follow and apply any standards of practice and competence maintained by his/her particular practice area(s)?** | | | |
| Yes | No | | |
| 1. **Do all medical team members, including dietitians, attend or participate in any continuing professional education inside or outside the country of Saudi Arabia?** | | | |
| Yes | No | | |
| **B. NUTRITION & DIETETICS SERVICES CARE IN THE CENTER** | | | |
| 1. **Does the centre provide nutrition & dietetics services, counselling, or has any Nutrition Specific Care?** | | | |
| Yes | No | I do not know | |
| 1. **If yes, who is the source of nutrition information?** | | | |
| Physicians | | | |
| Health Educators | | | |
| Dietitians | | | |
| Nurses | | | |
| Other, Specify ………. | | | |
| 1. **Provided nutrition & dietetics services and counselling, including:** | | | |
| Nutrition for specific age group | | | |
| Nutrition for pregnancy & lactation | | | |
| Weight problems (Obesity & overweight) | | | |
| Eating disorders | | | |
| Anaemia | | | |
| Growth and development | | | |
| Diabetes Mellitus | | | |
| Cardio Vascular Diseases | | | |
| Bone health problems | | | |
| Oncology | | | |
| Kidney problems | | | |
| Communicable diseases | | | |
| Gastroenterology problems such as food poisoning | | | |
| Acute problems | | | |
| Other, Specify ………. | | | |
| 1. **Within the organizational structure of your PCC, is the dietitian or nutrition/dietetics services part of it?** | | | |
| Yes | No | | |
| 1. **Does the dietitian or nutrition/dietetics services follow any food and nutrition-related legislation, regulations, standards and guidelines to practice?** | | | |
| Yes | No | | |
| 1. **If yes, are they international or national based standards?** | | | |
| International | National based standards | | |
| Please specify the name of the organization/system followed? | | | |
| ……………………………………………………………………………. | | | |
| 1. **Does the centre use current technology in clinical practice, including but not limited to: software, multimedia, webcasts, e-mail, instant messaging, file transfers, video conferencing, and electronic charting?** | | | |
| Yes | No | | |
| **C. DIETITIANS OF THE CENTRE** | | | |
| 1. **Are there any nutritionists/dietitians working in your PCC?** | | | |
| Yes | No | | |
| If yes, how many of them? | | | |
| …………………………………………………………………. | | | |
| 1. **If yes, are all working with your PCC clinical dietitians?** | | | |
| Yes | No | | |
| If not, please specify the number of clinical dietitians. | | | |
| …………………………………………………………………… | | | |
| 1. **If yes, are they all accredited by the Saudi Commission for Health Specialties (SCFHS) as Clinical?** | | | |
| Yes | No | | |
| 1. **Are all dietitians in your PCC members of the Saudi Dietetic Association?** | | | |
| Yes | No | | |
| 1. **What is the education level for dietitians working in the PCC?** | | | |
| BSc (How many ……….) | | | |
| Master (How many ……….) | | | |
| PhD (How many ……….) | | | |
| **D. ASSESSMENT OF NUTRITIONAL ASSESSMENT & ANTHROPOMETRY USED IN THE PCC** | | | |
| 1. **Does your PCC conduct any nutritional assessment for any age group as a prevention or treatment plan?** | | | |
| Yes | No | | |
| 1. **If yes, does the PCC use specific formats for nutritional assessment in general?** | | | |
| Yes | No | | |
| 1. **Does the PCC use specific formats for nutritional assessment for any age group in particular?** | | | |
| Yes | No | | |
| 1. **If yes, what are the tools used by the PCC for the nutritional assessment? (You can check more than one)** | | | |
| Diet history assessment | | | |
| Biochemical/ Hematological assessment | | | |
| Height including knee height & sitting height | | | |
| Physical assessment | | | |
| **Anthropometrics such as the following:** | | | |
| Weight | | | |
| Circumferences such as waist, MUAC… | | | |
| Skinfold thicknesses | | | |
| Body Composition Analyser | | | |
| Others, Please Specify ………………………………………………… | | | |
| 1. **Does the PCC use specific standards or reference data for assessment, such as anthropometric assessment?** | | | |
| Yes | No | | |
| 1. **If yes, please specify which reference data is used for comparison?** | | | |
| The wHO DatA | | | |
| The Saudi reference standards | | | |
| The CDC reference standards | | | |
| NCHS/WHO reference standards | | | |
| Others, Please Specify ………………………………………………… | | | |
| 1. **Are nutritionists/dietitians the only professionals performing assessments and taking anthropometric measurements, or do other medical team members perform them?** | | | |
| Dietitian Only | | | |
| Other Professionals Only | | | |
| Dietitians & other professionals | | | |
| 1. **Do you think your centre's nutrition & dietetics practices and services are in accordance with the national & international practice guidelines and would not need to be improved?** | | | |
| Yes | No | | |
| 1. **If yes, do you think that factors facilitating the use and the excellent nutrition & dietetics practising and servicing are?** | | | |
| The prior experience in dietetics and/or other health professional | | | |
| The provision of suitable assessment tools and machines | | | |
| The availability of clear procedures and/or protocols for this practice | | | |
| All the above choices are required to strengthen the practice. | | | |
| 1. **If not, do you think that it needs to be strengthened by:** | | | |
| Involvement of nutrition & dietetics professionals only to take part in nutrition Services. | | | |
| Providing more training to other health professionals to enhance their nutrition will support the system. | | | |
| Both choices are required to strengthen the practice. | | | |
| 1. **Do you see obese patients?** | | | |
| Yes | No | | |
| 1. **If yes, do you follow the Saudi Guidelines on preventing and managing obesity in your practice?** | | | |
| Yes | No | | |

**Section B: The Results of the 2016 Conducted Survey**

The study's results (**Table B)** showed a comprehensive evaluation of the provided nutrition care services at 18 PCCs in 2016. This study used interviews and onsite visits for data collection from the selected PC settings.

Findings showed that while PCCs provide nutrition management for a few nutrition-related diseases, no formal nutrition care services are assigned for all PCCs. In all centres, there are no RDs involved in nutrition care services. Health care providers in PC centres incoherently use nutrition care services. Nurses (27.8%) are the primary source of nutrition information, followed by physicians (27.8%). In the absence of dietitians, other PC providers assess patients’ nutritional status, measure anthropometry, and calculate body mass index.

Table B. The results of the 2016 survey

| **Variables name** | **FRQ** | **%** |
| --- | --- | --- |
| **Provided general, nutrition and dietetics services in PCCs** | | |
| **What is the role of your (PCC)?** |  |  |
| - Health promotion and disease prevention | 4 | 22.2 |
| - Intervention and treatment | 0 | 0.0 |
| - Both | 14 | 77.8 |
| - I do not know | 0 | 0.0 |
| **Do you provide community-based services outside your PCC?** |  |  |
| - Yes | 17 | 94.4 |
| - No | 1 | 5.6 |
| - I do not know | 0 | 0.0 |
| **Is your PCC accessed by the majority of:** |  |  |
| - Saudi | 8 | 44.4 |
| - Saudi & + Non-Saudi | 10 | 55.6 |
| **Which age group are the most accessed by your PCC?** |  |  |
| - Paediatrics (Infants, Toddlers, Children & Adolescents) - Adults (adults & elderlies) - All groups (all age groups served) | 5  2  11 | 27.8  11.1  61.1 |
| **Is each medical team member in the centre required to follow and apply any standards of practice and competence maintained by his/her particular practice area(s)**? |  |  |
| - Yes | 13 | 72.2 |
| - No | 5 | 27.8 |
| **Do all medical team members attend or participate in any continuing professional education inside or outside the country of Saudi Arabia?** |  |  |
| - Yes | 15 | 83.3 |
| - No | 2 | 11.1 |
| - Missing | 1 | 5.6 |
| **Does the centre provide nutrition & dietetics services, counselling, or has any Nutrition Specific Care?** |  |  |
| - Yes | 17 | 94.4 |
| - No | 1 | 5.6 |
| - I do not know | 0 | 0.0 |
| **If yes, who is the source of nutrition information?**   - Nurses - Physicians - Physicians, Nurses & Health educator - Social worker - Physicians & Nurses | 5  5  4  1  3 | 27.8  27.8  22.2  5.6  16.7 |
| **Provided nutrition & dietetics services and counselling, including:** | |  |
| - Nutrition for specific age group - Nutrition for pregnancy & lactation - Weight problems (Obesity & overweight) - Eating disorders - Anaemia - Growth and development - Diabetes Mellitus - Cardiovascular Diseases - Bone health problems - Oncology - Kidney problems - Communicable diseases - Gastroenterology problems such as food poisoning - Acute problems | SG  P&L  WP  ED  A  G&D  DM  CVD  BHP  O  KP  CD  GIT  AP | 50  83.3  61.1  22.2  50  66.7  94.4  38.9  22.2  11.1  33.3  16.7  38.9  11.1 |
| **Within the organizational structure of your PCC, is the dietitian or nutrition/dietetics services part of it?** |  |  |
| - Yes | 13 | 72.2 |
| - No | 5 | 27.8 |
| **Does the dietitian or nutrition/dietetics services follow any food and nutrition-related legislation, regulations, standards and guidelines to practice?** |  |  |
| - Yes | 8 | 44.4 |
| - No | 3 | 16.7 |
| - Missing | 7 | 38.9 |
| **If yes, are they international or national based standards?** |  |  |
| - International | 2 | 11.1 |
| - National-based standards | 8 | 44.4 |
| - Missing | 8 | 44.4 |
| **Does the centre use current technology in clinical practice, including but not limited to: software, multimedia, webcasts, e-mail, instant messaging, file transfers, video conferencing, and electronic charting?** |  |  |
| - Yes | 3 | 16.7 |
| - No | 14 | 77.8 |
| - Missing | 1 | 5.6 |
| **Dietitians in PCCs** | | |
| **Are there any nutritionists/dietitians working in your PCC?** |  |  |
| - Yes | 0 | 0.0 |
| - No | 17 | 94.4 |
| - Missing | 1 | 5.6 |
| **If yes, how many of them? ..................** |  | - |
| **If yes, how many of them are clinical dietitians and are they all accredited by the Saudi Commission for Health Specialties (SCFHS) as Clinical?** |  | - |
| **Are all dietitians in your PCC members of the Saudi Dietetic Association?** |  | - |
| - Yes |  | - |
| - No |  | - |
| **What is the level of education for dietitians who are working in PCC?** |  |  |
| - BSc (How many ...............................................) |  | - |
| - Master (How many ……………………..) |  | - |
| - PhD (How many ………………………..) |  | - |
| **Dietetic Practice (Assessment of Nutritional Status)** | | |
| **Does your PCC conduct any nutritional assessment for any age group as a prevention or treatment plan?** |  |  |
| - Yes | 11 | 61.1 |
| - No | 5 | 27.8 |
| - Missing | 2 | 11.1 |
| **If yes, does the PCC use specific formats for nutritional assessment in general?** |  |  |
| - Yes | 7 | 38.9 |
| - No | 9 | 50 |
| - Missing | 2 | 11.1 |
| **Does the PCC use specific formats for nutritional assessment for any age group?** |  |  |
| - Yes | 10 | 55.6 |
| - No | 6 | 33.3 |
| - Missing | 2 | 11.1 |
| **If yes, what are the tools used by the PCC for the nutritional assessment? (You can check more than one answer)** |  |  |
| - Diet history assessment |  | 29.4 |
| - Biochemical/ haematological assessment |  | 70.6 |
| - Height including knee height & sitting height |  | 76.5 |
| - Physical assessment |  | 23.5 |
| **Anthropometrics such as the following:** |  |  |
| - Weight |  | 100 |
| - Circumferences such as waist, MUAC… |  | 58.8 |
| - Skinfold thicknesses |  | 17.6 |
| - Body Composition Analyser |  | - |
| - Others, Please Specify: BMI |  | 11.8 |
| **Does the PCC use specific standards or reference data for assessment, such as anthropometric assessment?** |  |  |
| - Yes | 13 | 72.2 |
| - No | 1 | 5.6 |
| - Missing | 4 | 22.2 |
| **If yes, please specify which reference data is used for the comparison?** |  |  |
| - The wHO DatA |  | 80 |
| - The Saudi reference standards |  | 13.3 |
| - The CDC reference standards |  | 6.7 |
| - NCHS/WHO reference standards |  | 6.7 |
| - Others, Please Specify …………………………………………… | - | - |
| **Are nutritionists/dietitians the only professionals performing assessments and taking anthropometric measurements, or do other medical team members perform them?** |  |  |
| - Dietitian Only | - | - |
| - Other Professionals Only | 16 | 88.9 |
| - Dietitians & other professionals | - | - |
| - Missing | 2 | 11.1 |
| **Do you think your centre's nutrition & dietetics practices and services are in accordance with the national & international practice guidelines and would not need to be improved?** |  |  |
| - Yes | 8 | 44.4 |
| - No | 3 | 16.7 |
| - Missing | 7 | 38.9 |
| **If yes, do you think that factors facilitating the use and the excellent nutrition & dietetics practising and servicing are:** |  |  |
| - The prior experience in dietetics and/or other health professionals | 1 | 5.6 |
| - The provision of good assessment tools and machines | 1 | 5.6 |
| - The availability of clear procedures and/or protocols for this practice | - | - |
| - All the above choices are required to strengthen the practice | 3 | 16.7 |
| - Missing | 13 | 72.2 |
| **If not, do you think that it needs to be strengthened by:** |  |  |
| - Involvement of nutrition & dietetics professionals only to take part in nutrition Services | 4 | 22.2 |
| - Providing more training to other health professionals to enhance their nutrition will support the system | - | - |
| - Both choices are required to strengthen the practice | 8 | 44.4 |
| - Missing | 6 | 33.3 |
| *Responses from PCCs (n = 18) in 2016 survey.  *n (%) shows data presented as numbers and percentages. | | |
